# Removing the stumbling block of exosome applications in clinical and translational medicine: expand production and improve accuracy

**DOI:** 10.1186/s13287-023-03288-6

**Published:** 2023-04-01

**Authors:** Li Han, Zhirong Zhao, Chuanshi He, Jiami Li, Xiangyu Li, Man Lu

**Affiliations:** 1 Ultrasound Medical Center, Sichuan Clinical Research Center for Cancer, Sichuan Cancer Hospital & Institute, Sichuan Cancer Center, Affiliated Cancer Hospital of University of Electronic Science and Technology of China, Chengdu, 610041 Sichuan China; 2 College of Medicine, Southwest Jiaotong University, Chengdu, 610031 Sichuan China; 3 The School of Medicine, University of Electronic Science and Technology of China, Sichuan 611731 Chengdu, China

**Keywords:** Exosome, Optimization, Extraction, Translational medicine, Targeted delivery

## Abstract

Although the clinical application and transformation of exosomes are still in the exploration stage, the prospects are promising and have a profound impact on the future transformation medicine of exosomes. However, due to the limitation of production and poor targeting ability of exosomes, the extensive and rich biological functions of exosomes are restricted, and the potential of clinical transformation is limited. The current research is committed to solving the above problems and expanding the clinical application value, but it lacks an extensive, multi-angle, and comprehensive systematic summary and prospect. Therefore, we reviewed the current optimization strategies of exosomes in medical applications, including the exogenous treatment of parent cells and the improvement of extraction methods, and compared their advantages and disadvantages. Subsequently, the targeting ability was improved by carrying drugs and engineering the structure of exosomes to solve the problem of poor targeting ability in clinical transformation. In addition, we discussed other problems that may exist in the application of exosomes. Although the clinical application and transformation of exosomes are still in the exploratory stage, the prospects are promising and have a profound impact on drug delivery, clinical diagnosis and treatment, and regenerative medicine.

## Introduction

**Fig. 1:**
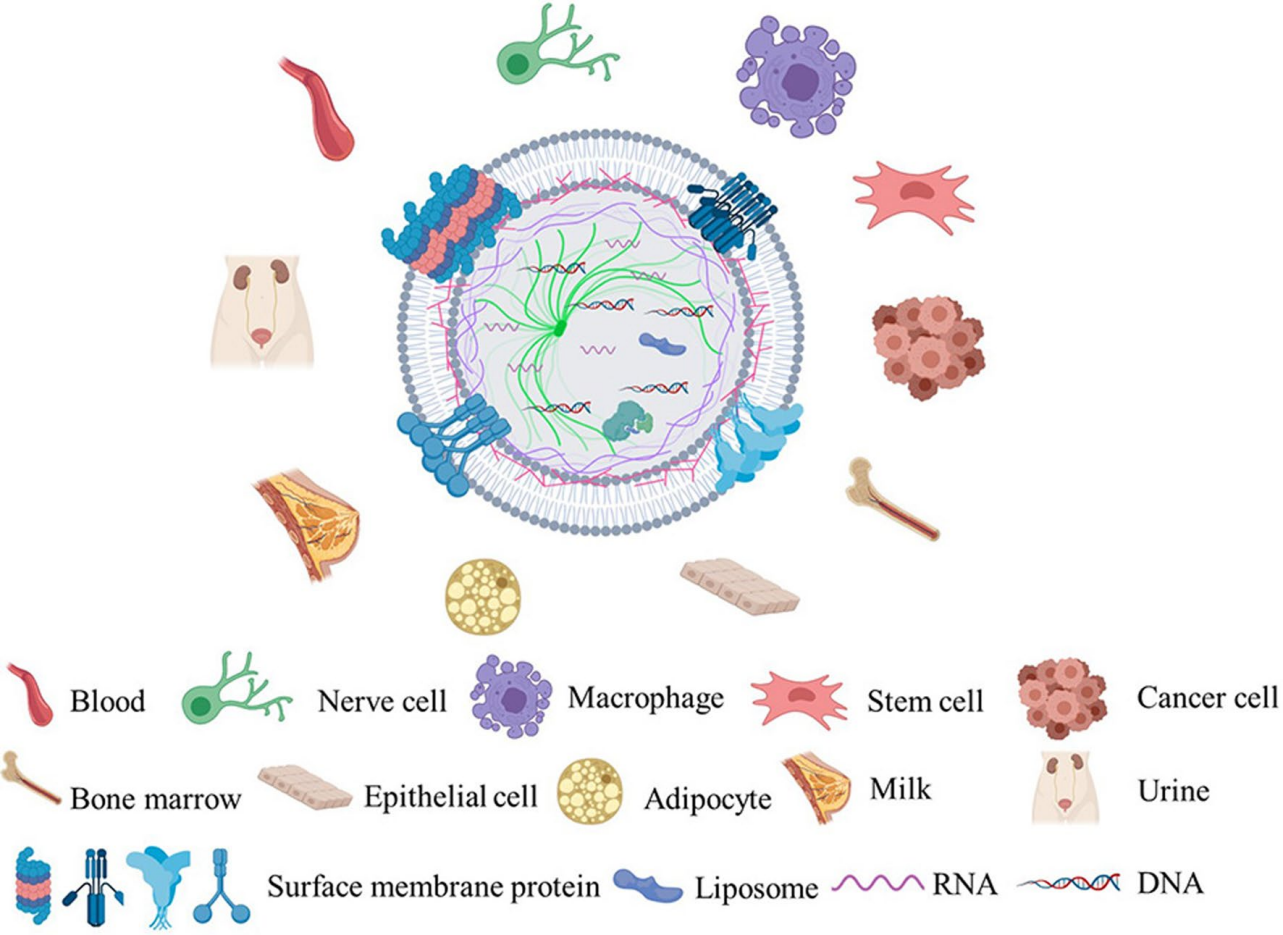
Origin and structural characteristics of exosomes

Extracellular vesicles (EVs) have received increasing attention as novel biomarkers, therapeutics, and drug delivery vehicles for diseases [1]. EVs can be classified according to their biosynthetic or release pathway, including exosomes with a diameter of 30–150 nm that originate from the endocytic pathway, microvesicles with a diameter of approximately 100–1000 nm that are directly released from the plasma membrane, and 50 nm–2 um that are generated by apoptosis [2]. Among them, the powerful advantages and functions of exosomes are highlighted in medical research and clinical applications due to their containing complex nucleic acids, RNA, and proteins. A variety of cells can secrete exosomes under normal and pathological conditions. Exosomes originate from the inward budding of the cell membrane to form an endosome. After undergoing the processes of forming a polycystic complex, directional assembly, and migration, the endosome fused with the cell membrane and excreted out of the cell by exocytosis [3, 4]. Exosomes have a topology similar to that of cells and contain proteins such as transmembrane protein (CD9, CD63, CD81), annexins, and heat shock proteins (HSP) [5–7]. And the exosomes were also enriched in cholesterol and sphingomyelin. Up to now, many studies have found that there are 41,860 kinds of protein, 2838 kinds of microRNA, and 3,408 kinds of mRNA in the exocrine body [8–10] (Fig. 1).

Exosomes not only mediate signal transmission between cells by binding to the plasma membrane, but also they are enriched in a variety of bioactive genes that can transmit messages between cells [11]. Furthermore, exosomes are present in all biological fluids. Exosomes extracted from body fluids were performed multicomponent analysis, which can accurately reflect the source of the cells in which they occur and their physio-pathological status [12]. It may assist in the diagnosis and prognosis of diseases. At present, with the gradual progress of research, exosomes have been paid more and more attention. For example, umbilical cord mesenchymal stem cell-derived exosomes (ucMSC-Exs) were validated in our previous study to promote pancreatic tissue repair in traumatic pancreatitis injury by inhibiting pancreatic acinar cell apoptosis and controlling the systemic inflammatory response [13]. Kong et al. [14] investigated the effect of cancer cell-derived exosomes linc00313 on M2 macrophage differentiation in non-small cell lung cancer (NSCLC). They found that cancer cell-derived exosomes linc00313 promoted M2 macrophage differentiation in non-small cell lung cancer by upregulating STAT6. Wang et al. investigated the efficacy and mechanism of epidermal stem cell-derived exosomes (ESCs-exo) in improving impaired diabetic wound healing, elucidating that ESCs-exo enhanced the proliferation and migration of diabetic fibroblasts and macrophages and accelerated M2 macrophage polarization to promote diabetic wound healing. Besides cell-derived exosomes, some natural exosomes from body fluids also affect important biological functions. Pan et al. [15] demonstrated that exosomes derived from urine stem cells (USCS) can induce neurogenesis and contribute to recover the function of cerebral ischemia. Mi et al. [16] found that saliva-derived exosomes can induce human umbilical vein endothelial cell (HUVEC) proliferation, migration, and angiogenesis in vitro and promote skin wound healing in vivo. Zhou et al. [17] found that human milk-derived exosomes ameliorated hyperoxia-induced cell injury to prevent neonatal bronchopulmonary dysplasia by inhibiting the downstream of the IL-17 signaling pathway. However, the bottleneck for exosomes to exert their extensive and abundant biological functions is limited yield and poor targeting ability. Therefore, it is an urgent problem to massively improve the yield of exosomes and their targeted enrichment in target organs [18–20].

Firstly, the multiple biological functions of exosomes depend on the accumulation of a certain amount of exosomes in target organ tissues. The improvement of exosome yield is a big problem for future applications. Extraction of exosomes is difficult due to their differences in size, content, function, and source [21, 22]. The current separation techniques cannot separate it accurately, resulting in relatively low exosome extraction purity and extraction yield [23, 24]. Therefore, how to efficiently enrich exosomes is an important topic at present, which is crucial for analyzing the molecular mechanism of the role of exosomes.

Secondly, another major obstacle to the future clinical application of exosomes is targeted delivery [25]. Improving the enrichment or colonization content of exosomes in target organs or target tissues can more efficiently exert the dominant effect of the exosomes and save the amount of the exosomes used for exerting the same effect, which will relieve the pressure for urgently improving the yield of the exosomes from the side. Natural exosomes are unable to deliver drugs efficiently and be targeted for applications due to several disadvantages such as poor stability and easy destruction [5]. In addition, after the exosomes enter the body, the utilization rate significantly decreases owing to blood circulation, immune clearance and retention of organs [26, 27]. To meet research needs, engineering exosomes by means such as the surface modification or drug loading is a way of practical application that makes exosomes superior in terms of yield and targeted therapy, thereby achieving a precise treatment of exosomes and accelerating the clinical application of exosomes [28, 29].

In this review, we first introduced strategies to solve the mass production problems of exosomes in clinical and research applications: exogenous treatment for parent cells and different extraction methods. Secondly, two methods of improving the targeting ability of exosomes are introduced. One is to improve the targeting ability of exosomes carrying drugs; the other is to improve the targeting ability by engineering modification of the exosome structure. Finally, we summarize the trends and possible challenges in future research and application of exosomes. Therefore, many researchers have made a lot of efforts how to standardize the isolation, purification, and quantification of exosomes and to improve the targeting ability of exosomes, which has laid the theoretical and technical foundation for the clinical application of exosome therapy.

## Optimization strategy of exosomes in clinical application and medical research—improving the yield of exosomes

### Exogenous treatment of parent cells

In the clinical application of medical research, the exosomes secreted by cells through paracrine function play a major role [30, 31]. Therefore, the status, vitality, proliferation status and living environment of parent cells are closely related to the content and function of exosomes [32, 33]. In order to produce safer and more effective exosomes for clinical transformation research on a large scale, different measures are taken for parent cells to change and affect the exosomes yield from the source, which is crucial for the development of new therapies for diseases in the future. We summarize recent advances regarding the exogenous management of parental cells.

#### Origin and cell status of parent cells

A variety of cells in the body can secrete exosomes, and the content of exosomes produced by different cell sources is also different. Some scholars have compared the production and doubling times of exosomes of mesenchymal stem cells from different sources. They have found that umbilical cord mesenchymal stem cells grow faster than mesenchymal stem cells from bone marrow or other tissues under the same culture conditions. Umbilical cord mesenchymal stem cells produce more and larger exosomes than bone marrow or other tissues [34]. Cell state is closely related to cell culture generation. Telomerase activity is continuously decreased during cell division and proliferation, resulting in cell telomere shortening and partial gene loss at the late stage of DNA replication. After multiple passages, the cell senescence occurs, and the differentiation ability of stem cells is gradually reduced. Similarly, the yield and purity of exosomes can be significantly reduced, resulting in an impact on the efficacy of exosome therapy. Some studies have found that there is a positive correlation between the degree of cell aging and cell generation by analyzing the biological characteristics of each generation of bone marrow mesenchymal stem cells. During the culture, the number of apoptotic cells increased significantly from the sixth generation. After the eighth generation, more apoptotic cells appeared and such aging phenomena as cytoplasmic vacuolation and cell body enlargement [35]. In addition, cell inoculation density may affect exosome secretion. Studies have shown that cell-to-cell contact can lead to various physiological changes in cells. This may include cessation of cell growth and changes in differentiation status [36]. One study reported that with the increase in the number of passages of mesenchymal stem cells, the biological activity of exosomes of mesenchymal stem cells was significantly reduced. They further demonstrated that reduced cell seeding density in the culture flask resulted in increased exosome production [37].

Therefore, scholars should pay attention to the following points when obtaining exosomes: First, select appropriate parent cells according to the needs of the experiment. When there are many kinds of parent cells to choose, the cell line with a high yield should be given priority. Secondly, we should select the parent cells with high activity and primitive to improve the ability to derive exosomes. Finally, frequently observe the cell density and growth state in the process of cell culture and select the parent cells in the logarithmic growth period to extract the exosomes.

#### Three-dimensional cell culture

Three-dimensional cell culture is a technique in which biological cells can be grown in artificially created three-dimensional spaces. Three-dimensional cell culture enables cells to migrate and grow in the three-dimensional tridimensional spatial structure of the vector, constituting three-dimensional cell carrier complexes and allowing cells to grow in all directions in vitro, similar to how they grow in vivo. Three-dimensional culture technology not only preserves the material and structural basis of the in vivo cellular microenvironment, but also exhibits the advantages of intuitiveness and condition controllability of cell culture. Studies have shown that the production of exosomes secreted by umbilical cord-derived mesenchymal stem cells based on three-dimensional culture is 20 times that of two-dimensional (2D) culture [34]. Zhang et al. found that the exosomes produced by three-dimensional cultured mesenchymal stem cells had a stronger anti-inflammatory effect and more stable properties by replacing the traditional two-dimensional culture system with the three-dimensional system [38]. Yang et al. cultured human umbilical cord mesenchymal stem cells using the three-dimensional culture technique and isolated exosomes from the obtained supernatant. They found that three-dimensional cultured exosomes (3D-Exo) can reduce Aβ production in Alzheimer's disease pathological cells and transgenic mice to improve memory and cognitive deficits in Alzheimer's mice [39]. Therefore, making full use of three-dimensional cell culture technology contributes to the large-scale production of exosomes and provides greater potential for the clinical transformation of exosomes.

#### Hypoxic preconditioning

Many diseases or injuries create a hypoxic microenvironment, while stem cells secreting exosomes have a natural tropism to the site of pathological changes such as hypoxia and inflammation and are able to regulate the secretion of corresponding substances depending on the microenvironmental conditions [40, 41]. Stem cell transplantation therapy holds promise for many difficult diseases, but the low number of surviving transplanted cells and their poor secretory function make it difficult to exert therapeutic utility [42]. Hypoxic preconditioning can effectively improve biological characteristics such as proliferative activity, anti-apoptotic ability, and secretory function of implanted cells. Studies have shown that hypoxic preconditions of mesenchymal stem cells (MSCs) can enhance their paracrine effects. This study, using an in vivo fracture model and in vitro experiments, confirms that hypoxic preconditioning can be used as a method to optimize the therapeutic effects of MSC-derived exosomes on fracture healing [43]. Therefore, hypoxic preconditioned stem cells can effectively optimize the yield and function of exosomes to exert better therapeutic effects.

#### Drug pretreatment

**Fig. 2:**
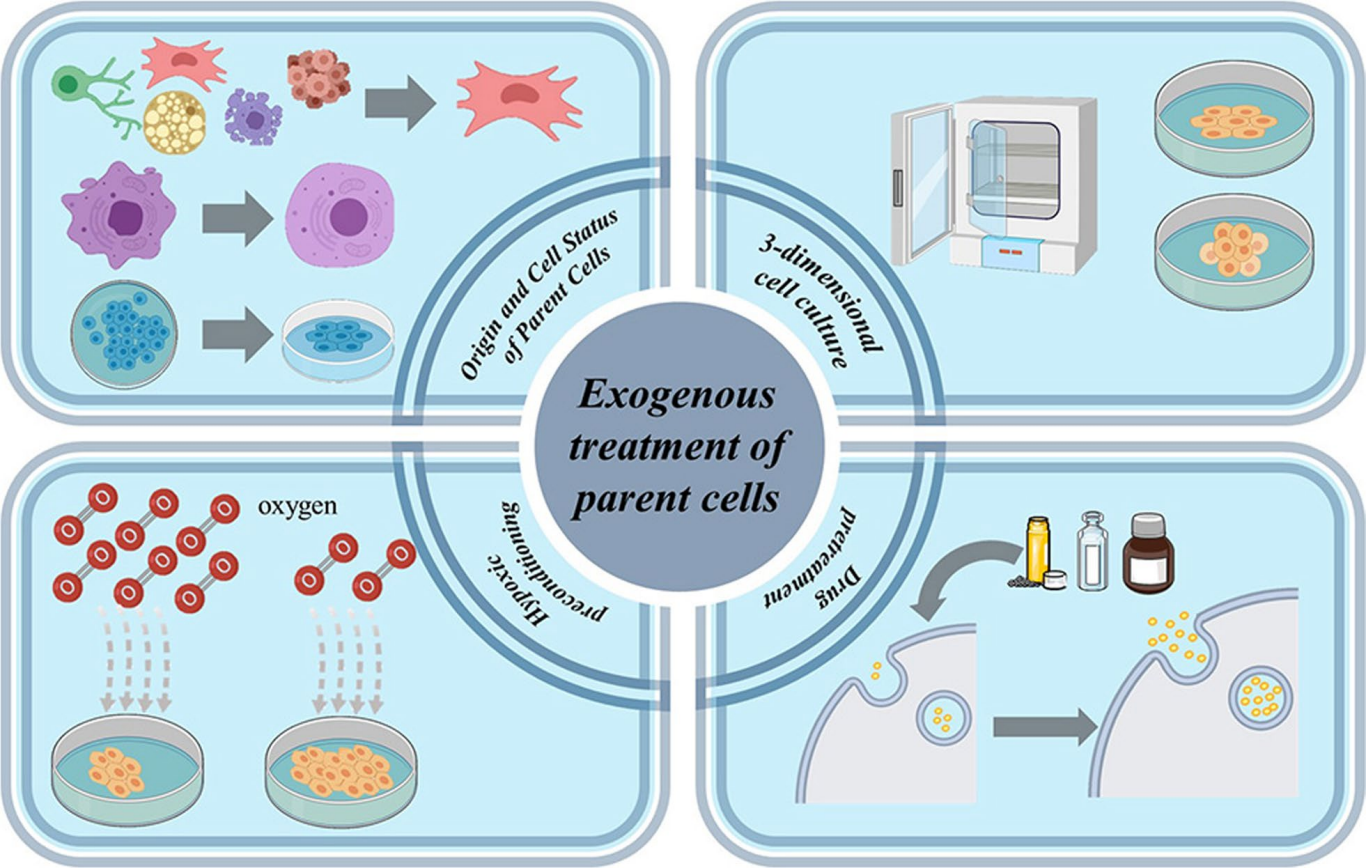
Improving the yield of exosomes from four aspects: origin and cell status of parent cells, three-dimensional cell culture, hypoxic preconditioning, and drug pretreatment

Because of the safety, low toxicity, and immunogenicity of exosomes, the therapeutic outcomes based on exosomes have greatly exceeded initial expectations in many clinically difficult diseases, but the yield of exosomes is a bottleneck for their widespread use. Taking different drug pretreatment to the parental cells could increase the yield of exosomes and optimize the function of exosomes to some extent. Studies have found that melatonin is synthesized from tryptophan and has protective effects under pathological conditions. As a novel preconditioning method, melatonin could effectively enhance the anti-oxidant, anti-inflammatory, and anti-apoptotic functions of exosomes in chronic kidney disease, diabetic wound healing, and ischemia–reperfusion therapy [44]. Another study pretreated bone marrow-derived mesenchymal stem cells (BMSCs) with traditional Chinese medicine Tongxinluo (TXL) and then collected their derived exosomes, verifying that exosomes secreted by TXL pretreated bone marrow-derived mesenchymal stem cells exhibited enhanced anti-apoptotic and anti-inflammatory effects in acute myocardial infarction (AMI) compared with untreated exosomes [45]. Therefore, proper pretreatment of the source cells combined with a suitable centrifugation method is effective in increasing exosome yield as well as functional properties (Fig. 2).

### Improving the yield of exosomes–extraction method

With the deepening of research in the field of exosomes, in order to better its biological function and further explore its molecular mechanism. Researchers have established many separation techniques to continuously optimize the extraction of exosomes (Table 1). Heretofore, there is still no method that can guarantee the content, purity, and biological activity of exosomes at the same time.

**Table 1:** Extraction method of exosomes

Methods	Definition	Advantages	Disadvantages	Years	References	
Ultracentrifugation	The method of separating and preparing exosomes by powerful centrifugal force in ultracentrifuges	Low cost-effectiveness, no risk of contamination, and the ability to extract larger amounts of exosomes	Consumes time, the extracted exosomes may be damaged due to high-speed centrifugation	2022	[55, 56]	Traditional methods
Ultrafiltration centrifugation	Ultrafiltration membranes with different relative molecular masses for selective separation	Imple, efficient, and does not affect the biological activity of exosomes	Exosomes may block the filter holes, resulting in the shortened life of the membrane and low separation efficiency,decreased yield	2022	[57, 58]	
Chromatography	The selective partitioning of different substances in different phase states to elute as a mixture in mobile relative stationary phases	Short time, high yield, and no need for special equipment	Low exosome purity as the ultracentrifugation method, exosome loss due to possible membrane adhesion	2018, 2022	[58, 59]	
Precipitation	The product of interest or major impurities in solution are isolated by precipitation in the form of an amorphous solid phase	Economical and does not need additional equipment	Low purity and recovery rate, more foreign proteins, uneven particle size, and destruction of exosome	2021	[60]	
Immunoaffinity chromatography	The highly specific affinity properties of antibodies to an antigen to separate a target from a mixture	Accurately separate the specified exosomes with high purity	High economic cost and low yield of exosomes	2021	[61]	
Immunomagnetic beads	Incubating magnetic beads coated with anti-marker antibodies with exosome vesicles	High specificity, simple operation, and no influence on the shape integrity of exosomes	Efficiency is low, and the biological activity of exosomes is easily affected by pH and salt concentration	2017, 2019	[6, 62, 63]4	
Biochips	Microchip technology based on the principle of specific interaction between molecules	Short time, accurately separate the specified exosomes with high purity	High economic cost, difficult clinical popularization, and low social benefit	2017	[65]	Emerging methods
Extraction kit	Exosomes are isolated by sedimentation by centrifugation	Large number of exosomes, convenient operation, simple, and fast	Quality control of extraction kits, high economic cost, and low social benefit	2019	[66]	

#### Traditional methods

The traditional extraction methods for exosomes are mainly as follows: ultracentrifugation, ultrafiltration centrifugation, chromatography, precipitation, immunoaffinity chromatography, and immunomagnetic beads. The advantages of ultracentrifugation are low cost-effectiveness, no risk of contamination, and the ability to extract larger amounts of exosomes [46]. But this method consumes time due to continuous centrifugation [47]. At the same time, the extracted exosomes may be damaged due to high-speed centrifugation. Ultrafiltration centrifugation uses ultrafiltration membranes with different relative molecular masses for selective separation [48, 49]. Small molecular substances are filtered to the other side of the membrane, while high relative molecular mass substances larger than the pore size of the membrane is retained on the ultrafiltration membrane. This method is simple, efficient, and does not affect the biological activity of exosomes. However, the disadvantage is that the exosomes may block the filter holes, resulting in the shortened life of the membrane and low separation efficiency. In addition, the exosomes remaining on the membrane may adhere, resulting in decreased yield. The greatest advantages of obtaining exosomes by chromatography are short time, high yield, and no need for special equipment. However, this method has the same problem of low exosome purity as the ultracentrifugation method. Secondly, exosome loss due to possible membrane adhesion during the operation is also a problem faced by this method [49, 50]. The advantage of the precipitation method is that it is economical and does not need additional equipment [51]. However, there are many problems with this separation method: low purity and recovery rate, more foreign proteins, uneven particle size, and destruction of exosomes. The advantage of the immunoaffinity chromatography is that it can accurately separate the specified exosomes with high purity [52]. However, the disadvantages of this method are high economic cost and low yield of exosomes. Exosomes have their specific markers (such as CD9, CD81, etc.). The exosomes can be adsorbed and separated by incubating magnetic beads coated with anti-marker antibodies with exosome vesicles. Because the heterogeneity of exosomes is consistent with their origin, the markers on different exosomes are also different. Different types of exosomes can be captured from samples by specific antibody combinations for selective separation. The magnetic bead method has the advantages of high specificity, simple operation, and no influence on the shape integrity of exosomes, but its efficiency is low, and the biological activity of exosomes is easily affected by pH and salt concentration, which is not conducive to downstream experiments and difficult to be widely popularized [53–55].

#### Emerging methods

In recent years, with the rapid development of science and technology, a number of new methods for the extraction of exosomes have emerged, such as biochips and extraction kits. There are more than 1000 electrodes in an alternating current electrokinetic (ACE) microarray chip device and the surface of which is coated with a thin layer of porous hydrogel. Exosomes are enriched in the high field area. The exosomes enriched at the chip electrode can be directly analyzed and identified by scanning electron microscope and immunofluorescence. The device can quickly separate and obtain exosomes from undiluted human plasma samples, and requires a small number of samples. It can concentrate the exosomes in the high-field area around the microelectrode within 15 min. Separation, washing, and chip fluorescence analysis can be completed within 30 min. However, the disadvantages of this method are high economic cost, difficult clinical popularization, and low social benefit [56]. The extraction kit method can obtain a large number of exosomes with complete structure and function from cell supernatant, serum plasma, or other body fluids by simple mixing and conventional centrifugation. This method has the advantages of convenient operation, simple experimental steps, fast extraction speed, and strong compatibility. It is widely applicable to the isolation of cell supernatant, serum, plasma, or other body fluid secretions. The disadvantages of this method are the same as alternating current electrokinetic (ACE) microarray chip device, and the quality control of the kit should also be considered [57].

Exosomes are widely distributed in various body fluids. Further studies are needed on how to simplify the extraction of exosomes, improve the production of exosomes, and how effectively and accurately separate exosomes. Different separation methods were chosen for different purposes and applications. Maybe a combination of several methods that can be selected simultaneously provides a more optimal strategy for effective exosome isolation.

## Optimization strategy of exosomes in clinical application and medical research–improve the targeting ability of exosomes

### Drug loading mode

Exosomes have similar biological structures and dominant physiological functions to those of the parent cells. However, exosomes are smaller and more stable. And exosomes contain abundant bioactive components such as nucleic acids, proteins, and lipids, carrying a lot of biological information. After exosomes are engulfed by target cells, intercellular signaling is completed by delivering these functional molecules to achieve functional regulation on recipient cells [58, 59]. However, the targeting of exosomes is insufficient. Therefore, it is easily cleared quickly and taken up by non-target cells after entering the body, or exosomes interact with the cell membrane, reducing the application of their dominant effect [60]. To solve the problem of limited targeting of exosomes, domestic and foreign scholars have modified the loading of the contents of the exosomes to make the exosomes have specific targeting or reduce the probability of being cleared by the organs, thereby achieving the purpose of improving the targeting and stability of the exosomes [61]. Exosome-targeted drug-loading strategies can be divided into two main categories, endogenous and exogenous.

#### Endogenous

Endogenous is the loading of various drug molecules (such as nucleic acids, viral proteins, and chemical drugs) in the interior of exosomes [62]. This method co-incubates parental cells with drugs or transfects drug encoding DNA or RNA to parental cells, allowing drugs to enter the cytoplasm. Drugs in the cytoplasm are sorted into exosomes in active or passive ways, and then drug-loaded exosomes can be obtained by suitable extraction methods. This method only involves the treatment and transformation of cells and hardly treats exosomes. Its advantage is that it keeps the integrity and functionality of exosomes, but its disadvantage is that the drug loading efficiency is relatively low. Pascucci et al. increased the uptake capacity of mesenchymal stem cells by passive diffusion of paclitaxel into the cells through the pretreatment of mesenchymal stem cells with paclitaxel. Exosomes were then extracted from mesenchymal stem cells co-incubated with paclitaxel. Finally, they found that exosomes extracted from mesenchymal stem cells co-incubated with paclitaxel had stronger anti-proliferative activity by comparing pre-treated exosomes with non-treated exosomes [63]. Chen et al. transfected human adipose mesenchymal stem cells with a lentivirus, enabling them to overexpress miR-375. Exosomes carrying miR375 were then extracted from human adipose mesenchymal stem cells in a rat model of skull defect. They found that the bone regenerative capacity of exosomes carrying miR375 was enhanced by comparison with exosomes extracted from untreated human adipose mesenchymal stem cells [64].

#### Exogenous

Exogenous loading means that exosomes are extracted first and then loaded with drugs to target specific cells or tissues for exerting effects. The advantages of exogenous are that the method is relatively simple and the drug-loading efficiency is relatively high. However, the drug-loading process may destroy the integrity of exosomes, and additional purification steps are required to remove unentrapped drugs. We mainly introduce two common exogenous methods including sonication and electroporation.

Sonication is the sonication of exosomes using a probe sonicator, which deforms the exosome membrane, creating transient small pores that increase the permeability of the membrane, thereby allowing small molecular substances to diffuse into the exosomes. The advantage of this method is the high loading efficiency. The disadvantage of this method is to damage the membrane of exosomes leading to exosome destruction. Haney et al. developed a novel exosome-based delivery system to load catalase into exosomes with the method of sonication. The sonicated exosomes have the advantage of high loading efficiency, sustained release of catalase, and protection from protease degradation. This method not only increases the targeted delivery of drug-loaded exosomes to target cells, but also increases the therapeutic effect of the drug [65]. Electroporation is a method to create temporary hydrophilic pore channels on the exosome membrane and increase the permeability of the exosome membrane so that small molecular substances can cross the pore channels to enter the interior of the exosome. The advantage of this method is the higher efficiency of loading, and the disadvantage is that electroporation causes exosome aggregation and destabilizes the exosome membrane. Wahlgren et al. loaded siRNA into exosomes by using electroporation. Plasma-derived exosomes are used as gene-delivery vehicles to transport exogenous siRNA to cells. Thus, exosomes effectively produce effects by targeted delivery of siRNA into monocytes and lymphocytes [66].

In clinical application and medical research, due to many factors, such as drug properties, disease types, and different cell types, it is necessary to design targeted drug delivery methods according to the different properties of loaded drugs and the characteristics of exosomes of different selected cells to improve the targeting ability of exosomes.

### Engineered exosome biomembrane

Exosomes are able to follow the blood circulation into various parts of the human body and even cross the blood–brain barrier. After exosomes enter the systemic circulation, what cannot avoid is engulfment or excretion by immune cells and the liver [67]. Therefore, their reaching target tissue efficiency depends on the degree of functionalization as well as the strength of interaction with the target cells. To improve the molecular transport capacity of exosomes and targeting properties, the engineering of exosomes is an effective approach. Engineered exosomes are gene fusions of ligands or targeting peptides to transmembrane proteins expressed on the surface of exosomes. Subsequently, donor cells are transfected with plasmids encoding fusion proteins that secrete engineered exosomes bearing targeting ligands on their surface, thereby conferring exosomes with cell and tissue specificity [68]. Methods used for engineering exosomes include chemical ligation of targeting peptides, genetic engineering to modify the exosome membrane, and magnetic nanoparticle technology. Lee et al. prepared sodium azide lipid-containing exosomes and conjugated them with targeting peptides using copper-free click chemistry to enhance targeting efficacy to cancer cells. They engineered the parental cells with membrane fusion liposomes, avoiding the destruction of the exosomes themselves while improving the targeting ability of exosomes in a more efficient and controlled manner [69]. Cheng et al. generated targeted exosomes of synthetic multivalent antibodies by modifying two different types of antibodies on the surface of exosomes. It elicits potent antitumor immunity in vitro and in vivo by expressing monoclonal antibodies specific for T-cell-associated CD3 and cancer cell-associated epidermal growth factor receptor (EGFR). This study achieved the ability to orient and activate cytotoxic T-cells for the targeted killing of cancer cells by engineering modifications to exosome surface membrane proteins [70]. Khongkow et al. utilized gold nanoparticles with multifunctional properties of surface modification combined with exosome membrane proteins for targeted delivery. After symbiosis with brain-targeted exosomes, the unique targeting properties of gold nanoparticles were demonstrated by their binding to brain cells under laminar flow conditions and their enhanced transport through the blood–brain barrier. Synthetic surface modification of gold nanoparticles with brain-targeted exosomes represents a very novel and efficient strategy to provide effective brain targeting [71].

**Fig. 3:**
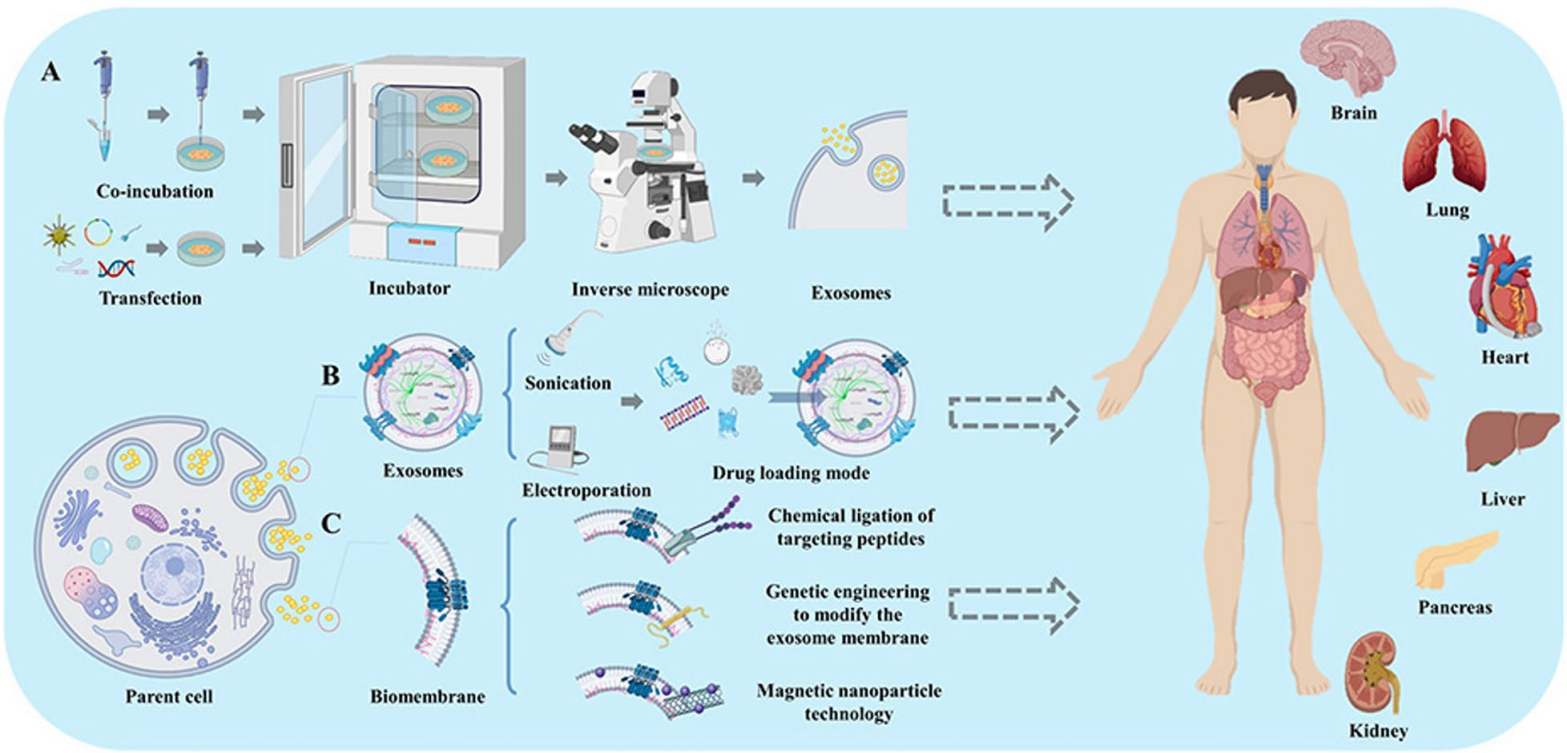
Improve the targeting ability of exosomes. **A**: two different endogenous loading modes, **B**: two different exogenous loading modes, **C**: three different engineered exosome biomembrane modes

The dominant effects of exosomes are mainly manifested in many processes, such as cell signaling, tissue transformation and re-differentiation, and regulation of signaling pathways. Exosomes are easily manipulated and target active molecules can be encapsulated during the production of exosomes secreted by parental cells and are able to cross multiple biological barriers (Fig. 3 and Table 2). Exosomes have weak targeting ability. This leads to the waste of exosomes in use and affects therapeutic efficacy. In addition, there is increased aggregation in non-target organs or tissues and toxic side effects. Therefore, improving the targeting ability of exosomes is an obligatory way to achieve a large number of future applications of exosomes.

**Table 2:** Methods for enhancing the targeting ability of exosomes

Modification strategies	Methods		Main contents	Years	References
Drug-loading strategies	Endogenous	Co-incubates parental cells with drugs	The parent cells were co incubated with the drug to make the drug enter the cytoplasm. The drugs in the cytoplasm are sorted into the exosomes through active or passive methods, and then the exosomes loaded with drugs can be obtained through appropriate extraction methods	2014	[72]
		Transfects drug encoding DNA or RNA to parental cells	The method of artificially introducing nucleic acid (DNA or RNA) into cells to change the characteristics of cells	2019	[73]
	Exogenous	Sonication	Sonication is the sonication of exosomes using a probe sonicator, which deforms the exosome membrane, creating transient small pores that increase the permeability of the membrane, thereby allowing small molecular substances to diffuse into the exosomes	2015	[74]
		Electroporation	Electroporation is a method to create temporary hydrophilic pore channels on the exosome membrane and increase the permeability of the exosome membrane so that small molecular substances can cross the pore channels to enter the interior of the exosome	2012	[75]
Engineered exosome biomembrane	Chemical ligation of targeting peptides		The chemical linkage of targeting peptides is to fuse relevant targeting peptides with exosomal highly expressed proteins to construct exosomes that specifically target various types of tissues or cells	2016	[78]
	Genetic engineering to modify the exosome membrane		Gene engineering fuses the gene sequence of the guiding protein or polypeptide with the gene sequence of the selected exosome membrane protein, which can effectively display the specific guiding peptide and protein on the exosome surface	2018	[79]
	Magnetic nanoparticle technology		Magnetic nanoparticle technology uses magnetic nanoparticles with dual targeting function to capture and release endogenous exosomes to target organs	2019	[80]

## Summarization and prospect

In recent years, exosomes have been found to serve as a novel kind of information carrier for intercellular information transmission, participating in the regulation of molecular and genetic characteristics of normal or abnormal cells. The presence of exosomes has provided the possibility for cell-free based therapies and sparked a boom in research with strong advantages. In order to obtain higher-quality exosomes for clinical application. It is very important to use appropriate exosome extraction methods, culture conditions, and new engineering transformation technologies [72, 73]. At present, there are many kinds of exosome extraction methods, all of which have their own advantages. However, the exosomes obtained by any extraction methods are heterogeneous, and a single exosome may contain different amounts and kinds of bioactive substances. Second, the differences between exosomes from different sources are still unclear, and their functional differences have not been fully elucidated. Hence, they cannot meet research and clinical needs. However, both an ideal drug targeting system and the system of the engineered exosome targeting capabilities must ensure that it is nontoxic and nonimmunogenic, and it is also free of adverse effects in vitro and in vivo, which cannot increase its human body burden. Therefore, the application of exosomes still needs further research development.

There are some unsolved problems in clinical application and medical research of exosomes besides the above-mentioned obstacles. For example, the storage of exosomes is time-sensitive and affected by many factors [74]. It should be used as soon as possible after extraction. Therefore, it is of great research value and clinical application prospect to develop a new storage method to maintain the structure and biological function of exosomes. Secondly, most researches mainly focus on the exosomes derived by human stem cells, and the research on the advantages and benefits of plant-derived exosomes should be strengthened. Plant-derived exosomes do not involve ethical issues and are relatively safe. However, there are still many unexplored problems in the mechanism of plant-derived exosomes, which need further study. In summary, although clinically applicable exosome extraction methods are not uniform, engineering is still in the stage of exploration. But the future development of exosomes is still promising. Exploring more methods that can optimize the composition and function of exosomes is a novel and promising direction and has profound implications for the translational medicine of exosomes in the future.

## Conclusion

Exosomes have attracted much attention in recent years as nanoscale biomarkers mediate cellular communication. In order to make better use of multiple advantageous effects, research on improving the yield and targeting ability of exosomes has taken a blowout. This review summarizes the current optimization strategies for exosomes in clinical applications and compares their advantages and disadvantages. In addition, we also propose possible problems and the research prospects in the application of exosomes. Although there are still many challenges in the application of exosomes in disease treatment, however, with the advancement of medical technology, it is just around the corner to further explore the potential of exosomes in translational medicine and provide new ways to create effective clinical diagnoses and treatment strategies.

## Data Availability

Not applicable.

## Abbreviations

RNA: Ribonucleic acid
Hsp: Heat shock proteins
mRNA: Messenger RNA
ucMSC-Exs: Umbilical cord mesenchymal stem cell-derived exosomes
NSCLC: Non-small cell lung cancer
STAT6: Signal transducer and activator of transcription 6
ESCs-exo: Stem cell-derived exosomes
USCS: Urine stem cells
HUVEC: Human umbilical vein endothelial cell
DNA: Deoxyribonucleic acid
3D: 3-Dimensional
3D-Exo: 3D-cultured exosomes
MSCs: Mesenchymal stem cells
BMSCs: Bone marrow-derived mesenchymal stem cells
TXL: Tongxinluo
AMI: Acute myocardial infarction
ACE: Alternating current electrokinetic
EGFR: Epidermal growth factor receptor
MCM2: Minichromosome maintenance complex component 2
